# Effects of the Insect Growth Regulator, Novaluron on Immature Alfalfa Leafcutting Bees, *Megachile rotundata*

**DOI:** 10.1673/031.011.0143

**Published:** 2011-04-08

**Authors:** Erin W. Hodgson, Theresa L. Pitts-Singer, James D. Barbour

**Affiliations:** ^1^ Department of Entomology, Iowa State University, Ames, Iowa 5001 1 -3140; ^2^ USDA-ARS Bee Biology and Systematics Laboratory, Utah State University, Logan, Utah 84322; ^3^ University of Idaho, Parma Research and Extension Center, Parma, Idaho 83660

**Keywords:** pollinator, alfalfa seed, Rimon, egg mortality

## Abstract

Alfalfa leafcutting bees, *Megachile rotundata* F. (Hymenoptera: Megachilidae), are the most common pollinators of alfalfa in the Pacific Northwest. Reports from users of *M. rotundata* in Idaho, Utah and Colorado have indicated exceptionally poor bee return from fields treated with novaluron to control *Lygus* spp. Our goal was to evaluate novaluron toxicity to immature *M. rotundata* using two different possible mechanisms of exposure. One goal was to assess immature mortality via treating nectar-pollen provisions and adults with novaluron. Immature *M. rotundata* mortality in all novaluron provision dosing treatments was significantly higher than the water or blank controls, providing evidence that novaluron is toxic to progeny in nest cells. The mean cumulative frequency showed that more eggs and 1st–2nd instars died compared to older instars. Female *M. rotundata* nested similarly in field cages during the field cage experiment; however, there was greater immature mortality in cages where females were fed sugar-water + novaluron compared to sugar-water only. Although females provided adequate provisions, there was a low percentage of egg hatch and larval development when females ingested novaluron before mating and nesting. Novaluron was also present in egg provision of bees collecting resources from novaluron-sprayed plants. At least 84% of progeny died when the females were allowed to mate and nest 24 hours after a novaluron application. Novaluron could be contributing to poor bee return in alfalfa grown for seed. Timely insecticide applications to suppress *Lygus* spp. is an important consideration to improve ongoing bee health.

## Introduction

The alfalfa leafcutting bee, *Megachile rotundata* F. (Hymenoptera: Megachilidae), is a solitary, but gregarious, cavity-nesting bee. Female bees make nests by cutting leaf pieces for lining individual cells within a linear cavity. Nectar and pollen are gathered and packed into each cell as provisions for feeding the developing larvae. *M. rotundata* was introduced into North America from Eurasia in the 1930s (Stephen 1962), and was later recognized as an efficient pollinator of alfalfa, *Medicago sativa,* compared to the honey bee, *Apis mellifera* (Peterson et al. 1992). In the 1950–1960s, growers were successful at increasing alfalfa seed set by using permanent shelters with “bee nesting boards,” or premade cavities, near or in fields. In this way growers reduced overall production costs by managing their own bees instead of purchasing bees each season. Approximately 36.3 million kg of alfalfa seed are now produced in the United States annually (Mueller 2008), with *M. rotundata* being the most dependable pollinator in the Pacific Northwest and Canada (Richards 1984; Bosch and Kemp 2005).

Current pollinator management practices include removing bee nesting boards from shelters in the fall, extracting the diapausing prepupae from boards, and holding prepupae in cold storage over the winter (Richards 1984; Frank 2003). The phrase “bee return” is often used as a measure of reproductive success of *M. rotundata*. Growers in the United States have experienced less than optimal bee return (e.g., under 50%) in the last forty years, and are forced to supplement their *M. rotundata* stock with additional bees (Petersen et al. 1992) purchased by the gallon from Canadian suppliers. Two to five gallons of bees are required to maintain desired seed set with 2007–2009 prices ranging from $20-85/gallon (1 gallon = 10,000 live bees).

There are likely several reasons why *M. rotundata* are difficult to sustain in the United States. Environmental factors, such as wind, rain and temperature can greatly affect bee productivity (Lerer et al. 1982; Richards 1996). Bee shelters force individuals to be in a highly aggregated environment that is conducive to disease, predation and parasitism (Eves et al. 1980). The complex management of *M. rotundata* is critical for sustainability (Richards 1984, 1993), and some growers are not adopting best management practice recommendations for optimal overwintering survival (Kemp and Bosch 2000; Pitts-Singer and James 2009). Insecticide use in alfalfa grown for seed and nearby field crops also must be considered a factor that could potentially limit *M. rotundata* bee return (Thompson 2003).

Although acute oral and contact screening of pesticide toxicity to *M. rotundata* is not required for registration as it is for *A. mellifera*, limited information on toxicity of pesticides to *M. rotundata* is available (EPA 1996; Starks and Banks 2003; Riedl et al. 2006). Adult *M. rotundata* may be less tolerant of pesticides than other bees due to greater surface-to-volume ratios (Johansen et al. 1983). Many insecticides are known to have lethal or sublethal effects on *M. rotundata* adults, especially if they are applied during bloom or during times of the day when bees are active (Riedl et al. 2006). Although insecticide labels may provide warnings to avoid applications during bloom, *M. rotundata* shelters cannot be moved during the flight season as can *A. mellifera* hives. The actual seasonal exposure risk to all life stages may not be accurately reflected in field and laboratory studies.

*Lygus* spp. (Hemiptera: Miridae) are the most economically damaging insect to alfalfa seed in Idaho and northern Utah (Hodgson and Pace 2007). Recently, several new chemistries have been released for *Lygus* spp. suppression (i.e., novaluron, acetomiprid, and flonicamid) and are promoted for use in a rotational resistance management program (Ishaaya et al. 2003). In 2007, several anecdotal reports from growers using *M. rotundata* to pollinate alfalfa seed fields in Idaho and Utah indicated exceptionally poor bee return from fields treated with novaluron to control *Lygus* spp. Novaluron (1-[3-chloro-4-(1,1,2-trifluoro-2-trifluoromethoxyethoxy) phenyl]-3-(2,6-difluorobenzoyl) urea) (Rimon 0.83 EC, Chemtura Corporation) is an insect growth regulator that disrupts cuticle formation and prevents molting. Novaluron acts by contact or ingestion, and as with other insect growth regulators, targets immature life stages. Tests for effects of novaluron and other chitin synthesis inhibitors on worker bumble bees, *Bombus terrestris*, revealed high egg and larval mortality (Mommaerts et al. 2006). There is growing concern that novaluron is causing high *M. rotundata* mortality under certain circumstances in alfalfa seed production.

There are several possible mechanisms of novaluron exposure to protected eggs and larvae (e.g., treated leaf pieces, tainted provisions, transovarial transmission). This article presents three experiments conducted to evaluate novaluron toxicity to immature *M. rotundata*. The first and second are lab and field experiments designed to assess mortality by applying novaluron to nectar-pollen provisions and adults. The third experiment measured novaluron concentrations in provisions. This work serves as a precursor for future research that defines insecticide exposure routes in the field.

## Materials and Methods

### Provision dosing

On four dates (18 July, 24 July, 31 July, and 8 August) in 2008, *M. rotundata* eggs were collected from paper straws that had been inserted into bee nesting boards from novaluron-free alfalfa seed fields in Cache County, Utah. Individual eggs and their provisions were obtained by dissecting paper straws filled with linear cavities, and by removing leaf-disc caps covering the openings with a forceps. Intact cells, minus the cap, with provisions and containing a *M. rotundata* egg were individually placed into 96-well culture plates. To ensure that only eggs were used (Abbott et al. 2008), a stereomicroscope was used to distinguish eggs from first instars within the chorion. Intact *M. rotundata* eggs are shiny, smooth, and show no segmentation (Trostle and Torchio 1994).

The currently recommended novaluron (Rimon 0.83EC) field rate for *Lygus* spp. is 12 fl/oz per acre (745 ml/ha) using 30 gal/water per acre (238 liters/ha); this dilution is equal to 3 µl of Rimon 0.83EC in 1 ml water. A stock solution of Rimon 0.83EC treatment was prepared in distilled water just prior to application to avoid product degradation. Four novaluron treatments were applied to *M. rotundata* provisions: 1) 10 times the field rate, 2) 2 times the field rate, 3) the field rate, 4) one-half the field rate. Two controls were also used: an untreated water control [water], and a blank treatment.

Egg availability varied across collection dates. Treatments were applied to pollen-nectar provisions with eggs on each collection day. Each treatment was applied to 96 eggs for the 18 July and 31 July collections. The 24 July and 8 August collections had 84 eggs per treatment. Treatments 1–5 received a 1 µl droplet of the respective dilutions with a 50 µl repeating dispenser syringe (Hamilton PB600, www.hamiltoncompany.com). Droplets were placed on top of the provision, approximately 1mm away from the side of the egg. Separate syringes were designated for each treatment to avoid contamination.

Well plates were covered and incubated at 27° C in 24-hour darkness for 21 days. Samples were visually evaluated for egg hatch and larval development every 2–4 days using a stereomicroscope. Development to the prepupal stage, where larvae spin a silken cocoon, was the criteria for survival (Guirguis and Brindley 1974). Prepupae were not monitored for further development to pupal or adult stages. Observed mortality was assigned to one of four categories: 1) larvae never hatched [egg], 2) dead, first and second instars [1–2], 3) dead, third and fourth instars [3–4], 4) dead, fifth instars [5]. Instars were identified according to Trostle and Torchio (1994).

The effect of treatment on the number of dead eggs and larvae was assessed using a generalized linear mixed model in a randomized block design where collection dates were blocks, with a binomial distribution and logit link (GLIMMIX, SAS/STAT 9.1.3 SAS Institute Inc.). The mean percent mortality of eggs and larvae was summarized by treatment to assess cumulative frequency.

### Adult dosing

In July 2008, four field cages (6.1 m × 6.1 m × 1.8 m) with fine mesh screening were erected over an alfalfa seed field at the Utah State University Greenville Research Farm in Logan, Utah. A small *M. rotundata* bee shelter with a removable nesting block was placed in the center of each cage, approximately 1 m off the ground with nesting holes facing southeast. Adult *M. rotundata* were obtained from incubation at the USDAARS Bee Biology Laboratory, Logan, UT. Adults were divided into two feeding treatments, 1) 10% sugar-water or 2) 10% sugar-water + the full field rate of novaluron (Rimon 0.83EC). Due to space and equipment limitations, we did not evaluate other novaluron concentrations. On Day 0, newly emerged males and females were separated into treatments and fed for 24 hours. On Day 1, adults (15 females and 20 males) were released in each cage and allowed to forage, mate and nest for 7–9 days. Cages 2 and 4 contained adults fed with sugar-water only and cages 1 and 3 contained bees fed sugar-water plus novaluron. On Day 7, the nesting block was removed and replaced. The experiment was repeated two more times; however, nesting during the second and third replications was substantially lower due to limited flower availability inside the cages, and no data were obtained.

**Figure 1.**
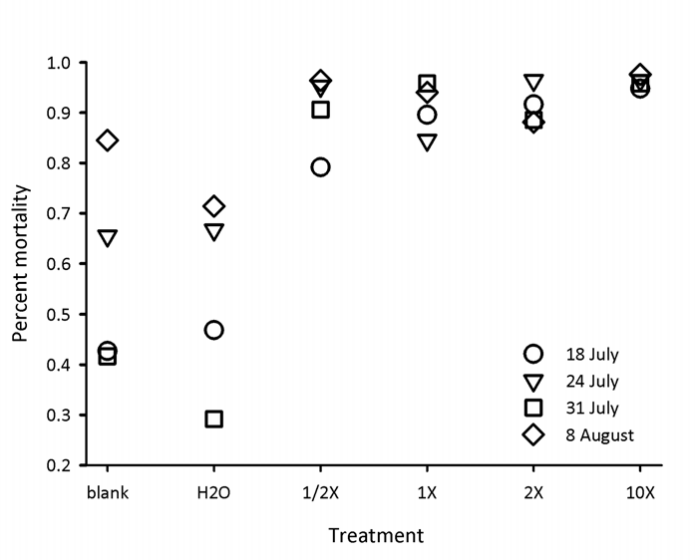
Mean percent mortality of *Megachile rotundata* provisions dosed with respective treatments by each block. Various concentrations of novaluron are indicated with an “X.” High quality figures are available online.

**Figure 2.**
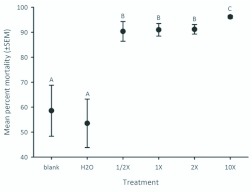
Mean percent mortality of *Megachile rotundata* reared on provisions dosed with respective treatments (±SEM). Various concentrations of novaluron are indicated with an “X.” Treatments with the same letter are not significantly different from each other (α = 0.05). High quality figures are available online.

The nesting straws containing bee cells were extracted from the nesting block and and analyzed using x-radiography (30 s exposure at 25kvp in Faxitron 43804N, Faxitron X-Ray, www.faxitron.com) (Stephen and Undurraga 1976). The progeny from each replicate were incubated at 27° C for 24-hour in darkness for 21 days. On day 28, a final x-ray was taken to estimate development. Leaf cells were then dissected to determine actual survivorship. Progeny were considered alive if they reached the prepupal stage. Percent mortality was calculated for all cages in all three replicates.

### Provision testing

In July 2009, four field cages (6.1 m × 6.1 m × 1.8 m) with fine mesh screening were used to cover an alfalfa seed stand at the Utah State University Greenfield Research Farm, Logan, Utah. A small *M. rotundata* bee shelter with a removable nesting block was placed in the center of each cage, approximately 1 m off the ground with a southeast orientation. Adult *M. rotundata* were obtained from incubation at the USDA-ARS Bee Biology Laboratory, Logan, UT. On day 0, the alfalfa in one of two paired cages was sprayed with the full field rate of novaluron using a hand sprayer. On day 1, 10–15 females and 15–20 males were released into each cage and allowed to forage, mate and nest for 6 days. On day 7, all nests and bees were removed from cages. If enough bloom was available as forage for bee nesting, then a second, and in one case a third, application of novaluron was applied to the same cage on the evening of day 7. The nests from the cages were examined using x-ray to determine the number of cells produced. If possible, some cells were removed for creating provision samples (provisions from at least 8 cells were required per sample), which were sent to the USDA-AMS-National Science Laboratory in Gastonia, NC for pesticide residue extraction. Other cells were kept for rearing as was done in 2008 to determine brood mortality for nests recovered from each cage. For reared bees, screening continued until bees became adults (non-diapausing bees) or diapausing prepupae (live larvae).

**Figure 3.**
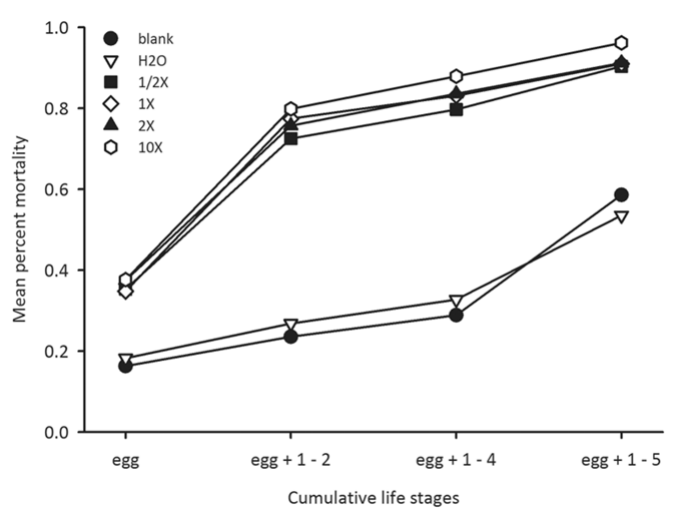
Cumulative mortality frequency of *Megachile rotundata* provisions dosed with respective treatments by life stage (where numbers indicate instar), cumulative mortality frequency of *Megachile rotundata* reared on provisions dosed with respective treatments. Various concentrations of novaluron are indicated with an “X.” Each point represents the total mortality over the indicated life stage. High quality figures are available online.

**Table 1.**
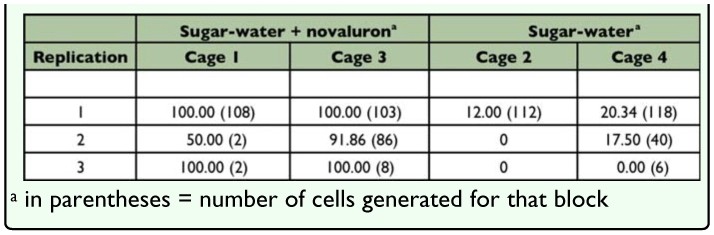
Percent progeny mortality from novaluron dosed and undosed adult *Megachile rotundata*

**Table 2.**
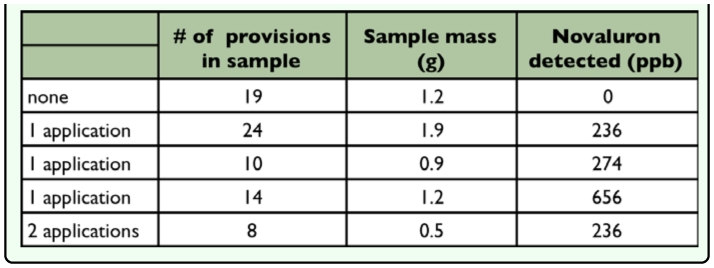
Amount of novaluron detected from *Megachile rotundata* provisions amassed by females foraging in novaluron-treated or untreated alfalfa

**Figure 4.**
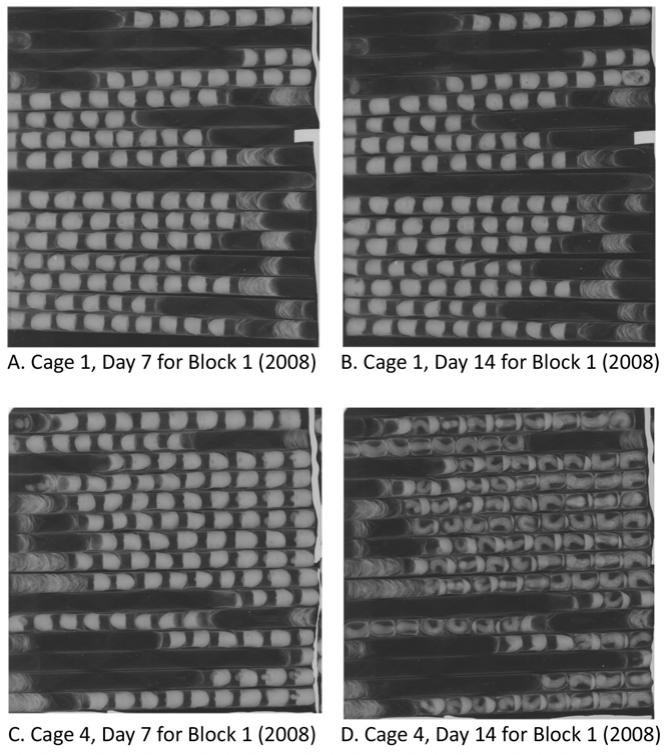
Example of x-rayed paper straws with *Megachile rotundata* cells. Note the absence of larval development after 14 days when adults are fed novaluron for 24 hours (B) compared to when adults are not exposed (D). High quality figures are available online.

## Results

### Provision dosing

Percentage mortality for all treatments is reported for each block (Figure 1). Mortality was significantly higher for all novaluron treatments compared to the blank and water treatments (*F* = 67.24; df =5, 15; *P* < 0.001; Figure 2). Among the novaluron treatments, mortality was significantly higher in the 10X treatment and did not differ among the 1/2X, 1X, and 2X treatments (Figure 2). The mean cumulative percentage mortality showed that more eggs and 1^st^–2^nd^ instars died compared to older larvae for all treatments (Figure 3).

**Figure 5.**
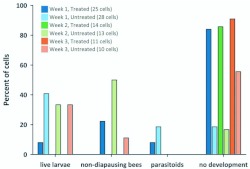
Destiny of *Megachile rotundata* progeny as determined from x-ray screening 28 days after nesting. Non-diapausing bees had already developed into prepupae or adults. High quality figures are available online.

### Adult dosing

For the first replication, there was 100% progeny mortality for the two cages where *M. rotundata* adults were fed sugar-water + novaluron (Table 1). These results can be compared to 12 and 20% progeny mortality for the two cages where adult *M. rotundata* were fed sugar-water (Table 1). Figure 4 demonstrates an example of larval development for the two treatments. In all four cages of the first replication, females produced similar numbers of eggs; however, in the sugarwater + novaluron treatment most were not viable (Table 1). Most of the progeny were dead eggs or young larvae.

### Provision testing

Immature *M. rotundata* mortality was also higher than expected when females were indirectly exposed to novaluron in field cages sprayed with the full field rate. At least 84% of progeny died when the females were allowed to mate and nest 24 hours after a novaluron application (Figure 5). There were five provision samples collected from the field cages in 2009 and tested for the presence of novaluron. The four provision samples from the novaluron-treated cages all had detectable levels (236, 236, 274, 656 parts per billion); whereas the provisions from the untreated cage had no detectable novaluron (Table 2). Unfortunately, there are no toxicity guidelines for mortality of novaluron to *M. rotundata*.

## Discussion

This research was designed to be the “worst case scenario” for maximum exposure of novaluron to immature *M. rotundata*. In commercial alfalfa seed production, we would not expect immatures to receive full field rates of novaluron. Because of its mode of action, it is not surprising that this insect growth regulator insecticide caused mortality to *M. rotundata* eggs and larvae. The high percent mortality at the 1/2X and 1X novaluron field rates was not anticipated. An LD50 for novaluron could not be estimated because mortality was >85% for all treatments (Figures 1, 2). We did not evaluate a lower concentration than the 1/2X rate because it would not likely provide effective suppression of *Lygus* spp. in commercial alfalfa grown for seed. By dosing *M. rotundata* provisions and female adults, we created situations for revealing the effect of novaluron on immature bees. Indeed, the bee loss in the commercial situations was similar to the high mortality in eggs and young larvae that we found in this study for novaluron exposure.

The data also confirmed potential avenues through which *M. rotundata* eggs and larvae can be exposed to novaluron. Egg exposure can occur transovarially; exposure of both eggs and larvae can occur through direct contact with novaluron-tainted provisions. Foraging female adults may come into cuticular contact with novaluron on treated alfalfa leaves or flowers parts, may ingest the novaluron when cutting leaf pieces for cellbuilding or when grooming their contaminated body parts, or may ingest nectar and pollen that contains novaluron. The absorbed or ingested insecticide might then be transferred to the eggs inside the female's body (Mommaerts et al. 2006). Females may also collect field-contaminated nectar and pollen that is used as the mass provision, although we believe that this scenario is unlikely because novaluron is not a systemic insecticide. The provision may become tainted if novaluron leaches into the provision from treated leaf pieces that form the bee cell or if the female regurgitates contaminated nectar while creating the mass. None of the experiments completely excludes any of these possible scenarios.

This tendency for death early in development is similar to observations made in 2007, and suggests that progeny are being exposed to novaluron shortly after egg deposition on the provision. At what point progeny are getting exposed to an insecticide could have major implications for mortality rates. The percent of dead, immature bees was higher than expected in the blank and water treatments, especially at later dates (Figure 2). Several experimental factors likely increased mortality, such as cell handling, incubation as separate cells without leaf caps, and dehydration. Naturally-occurring pathogens were a likely source of mortality in this experiment, especially for the older larvae. However, compared to the water and blank treatments, the novaluron-treated provisions had more dead eggs and young larvae than dead older larvae (Figure 3).

The immature bees on the treated provisions are likely to have made contact with the novaluron early in development. It is possible that the chemical diffused through the provision mass from the point of application to the egg and prevented it from molting, and, thus, prevented the larvae from hatching. It is also possible that older instars may have come into contact with the insecticide as it diffused through the provision. The consistency, moisture, and texture of *M. rotundata* provisions is highly variable (Pitts-Singer 2004), and could be a factor influencing timing of mortality for eggs and larvae. Although treatments were applied to the provision directly adjacent to the egg, there would likely be variability in the timing of first exposure to novaluron.

By feeding adult females in the laboratory or allowing them to forage on alfalfa on which novaluron was applied, strong evidence was obtained that when adult females ingest or come into direct contact with novaluron, they produce a high percentage of unviable eggs. These results are similar to those found for *B. terrestris* (Mommaerts et al. 2006) and *Tribolium castaneum* (Trostanetsky and Kostyukovsky 2008). We also found that by foraging on treated alfalfa, nesting females are capable of producing provisions with detectable levels of novaluron, ranging from 236–656 ppb. However, we cannot determine from this experiment whether eggs died because nonviable eggs were laid or whether eggs were killed by the novaluron present in the provisions.

Several questions about immature *M. rotundata* mortality still remain and should be examined in future research. Although significantly more mortality occurred at all of the four novaluron dose rates in provisions, we do not know why the eggs and larvae died. We also do not know if or how the novaluron was coming into contact and killing eggs and larvae (e.g., chorion absorption, ingestion, or both). This research evaluated egg and larval mortality and did not measure survivorship beyond the prepual stage. As a result, we are not able to describe sublethal effects on surviving adults that were exposed to novaluron as eggs. *M. rotundata* exposed to novaluron as immatures may not be healthy and productive and may indirectly contribute to poor bee returns the following year.

Because of the extremely high mortality rates discovered in this study, novaluron should be used with caution in alfalfa grown for seed where *M. rotundata* are used as pollinators. Although we did not dose provisions or adults of other bee species and beneficial insects, similar mortality rates may be expected. Growers and pollinating service companies should be aware of treating pests with insect growth regulating insecticides during active pollination in alfalfa. Therefore, timely application to suppress *Lygus* spp. and other economic pests is an important consideration for increasing bee return rates in alfalfa. Spraying insecticides before or after peak pollination and periods in the summer may still provide adequate pest control while limiting immature pollinator mortality.
